# Supervised Filter Learning for Representation Based Face Recognition

**DOI:** 10.1371/journal.pone.0159084

**Published:** 2016-07-14

**Authors:** Chao Bi, Lei Zhang, Miao Qi, Caixia Zheng, Yugen Yi, Jianzhong Wang, Baoxue Zhang

**Affiliations:** 1 College of Computer Science and Information Technology, Northeast Normal University, Changchun, China; 2 Changchun Institute of Optics, Fine Mechanics and Physics, CAS, Changchun, China; 3 School of Software, Jiangxi Normal University, Nanchang, China; 4 School of Statistics, Capital University of Economics and Business, Beijing, China; Jiangnan University, CHINA

## Abstract

Representation based classification methods, such as Sparse Representation Classification (SRC) and Linear Regression Classification (LRC) have been developed for face recognition problem successfully. However, most of these methods use the original face images without any preprocessing for recognition. Thus, their performances may be affected by some problematic factors (such as illumination and expression variances) in the face images. In order to overcome this limitation, a novel supervised filter learning algorithm is proposed for representation based face recognition in this paper. The underlying idea of our algorithm is to learn a filter so that the within-class representation residuals of the faces' Local Binary Pattern (LBP) features are minimized and the between-class representation residuals of the faces' LBP features are maximized. Therefore, the LBP features of filtered face images are more discriminative for representation based classifiers. Furthermore, we also extend our algorithm for heterogeneous face recognition problem. Extensive experiments are carried out on five databases and the experimental results verify the efficacy of the proposed algorithm.

## Introduction

Automatic face recognition has become a very active topic in computer vision and related research fields [1]. However, face recognition is still a very difficult task in practice due to the following two problematic factors. One is the appearance variations including facial expression, pose, aging, illumination changes, the other is the man-made variations, e.g. the noises from the cameras. The performances of many recognition approaches degrade significantly in these cases.

Recently, the representation based methods have been widely used in face recognition problem. In [2], Wright et al. proposed a sparse representation based classification (SRC) method for face recognition. SRC first sparsely codes a query face image by the original training images, and then the classification is performed by checking which class leads to the minimal representation residual of the query image. Later, Naseem et al. [3] proposed a linear regression based classification (LRC) method based on the assumption that patterns from the same class lie on a linear subspace, so the test image should be well represented as a linear combination of the training images from the same class. The main difference between SRC and LRC is the regularization they employed. That is, SRC utilizes the L_1_ norm regularization to make the representation coefficients to be sparse, while the L_2_ norm regularization is adopted in LRC to ensure the learning problem to be well posed. Since the experimental results in [2] and [3] demonstrated that SRC and LRC achieved impressive face recognition performances, the research of representation based face recognition was largely boosted and lots of approaches have been developed [4–8]. However, these representation based methods all utilized the original face image without any preprocessing for classification. Thus, as we have analyzed above, their performances may be affected by the problematic factors in face images.

Nowadays, various feature extraction approaches have been employed for face recognition. Among these approaches, Principal Component Analysis (PCA) [9], Linear Discriminant Analysis (LDA) [10] and their related extensions [11–15]have been well studied and widely utilized to extract low-dimensional features from the high-dimensional face images. However, since some recent studies have shown that high-dimensional face images possibly reside on a nonlinear manifold, many manifold learning methods such as Isometric Feature Mapping (ISOMAP) [16], Local Linear Embedding (LLE) [17], Laplacian Eigenmap (LE) [18] and their extensions have also been proposed for face recognition. Although the aforementioned feature extraction algorithms worked well, they all belong to the subspace based method and can only extract the holistic features of face images, which may lead them to be unstable to local variances such as expression, occlusion, and misalignment [19]. As a result, local descriptors such as Local Binary Pattern (LBP) have attracted more and more attention for their robustness to local distortions [20, 21]. The LBP operator [22] is a texture descriptor which describes the neighboring changes around each pixel. It has been successfully used in face recognition applications due to its invariance to the changes of illumination and expression in face images and computational efficiency. Considering the advantages of LBP in face recognition [23], many LBP variants have been proposed. In LGBP [24], GVLBP [25] and HGPP [26], instead of directly using the pixel intensity to compute the LBP features, multi-scale and multi-orientation Gabor filters were employed for encoding the face images. Then, the LBP histogram was obtained from the encoded images. Zhao et al. first extracted the gradient information from face image using Sobel operator and then applied LBP to the gradient images for feature extraction [27]. The LBP has also been adopted to extract the features for representation based classification techniques. In [28] and [29], some researchers combined LBP with SRC for face recognition. In their methods, the LBP features were first extracted from the face images. Then, the SRC was utilized for classification. Kang et al. employed LBP to extract local features of the face images so that the performance of kernel SRC could be improved [30]. In [31], Lee also used the Gabor-LBP features for face image representation in SRC.

Although the experimental results in [28, 29]indicate that the LBP can improve the performances of representation based face recognition techniques, a main drawback of these methods is that the label information is neglected during the local feature extraction of LBP, which may weaken their discriminative ability. In order to overcome this limitation, Lei et al. proposed an Image Filter Learning (IFL) method for face recognition [19]. In IFL, an image filter which can explore the discriminative information for face representation was first learned. Then, the LBP features were extracted from the filtered face images for recognition. However, IFL learns the discriminative image filter based on Fisher criterion. Thus, it may not be suitable for representation based face recognition methods in which the classification is determined by the representation residuals. Furthermore, the Fisher criterion may also make it not suitable to non-Gaussian distributed face images [32].

In this paper, a new supervised filter learning (SFL) algorithm is proposed to improve the discriminative ability of LBP features for representation based face recognition. Compared with other algorithms, our algorithm possesses two advantages. Firstly, different from LGBP [24], GVLBP [25], HGPP [26] and Sobel-LBP [27] in which the image filters are defined in an ad hoc way, the optimal filter in our algorithm is learned by a supervised data-driven manner. Therefore, the LBP features obtained in our algorithm are more discriminative than them. Secondly, unlike IFL [19] which learns the filter based on Fisher criterion, our proposed SFL is specially designed for representation based face recognition methods. That is, the main difference between IFL and the proposed algorithm is that the filter in IFL is learned by minimizing the within-class scatter and maximizing the between-class scatter of faces’ LBP features, while the filter in our algorithm is learned through reducing the within-class representation residual and enlarging the between-class representation residual of faces' LBP features. As a result, it can be seen from the experimental results on five benchmark face databases (Yale, AR, CMUPIE, LFW and VLNHF) that the performances of our algorithm are better than IFL and some other algorithms for representation based face recognition problem.

The remaining part of the paper is organized as follows: ‘Related Work’ section briefly reviews the LBP and IFL. 'The Proposed Algorithm' section describes the details of our algorithm. Experimental results and analysis are provided in ‘Experiments’ section and ‘Conclusions’ section gives the conclusion of this paper.

## Related Work

In this section, two related works including Local Binary Pattern (LBP) [33] and Image Filter Learning (IFL) [19] are briefly reviewed.

### Local Binary Pattern

**Fig 1:**
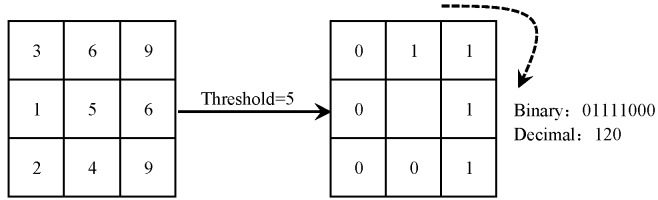
An example of LBP.

**Fig 2:**
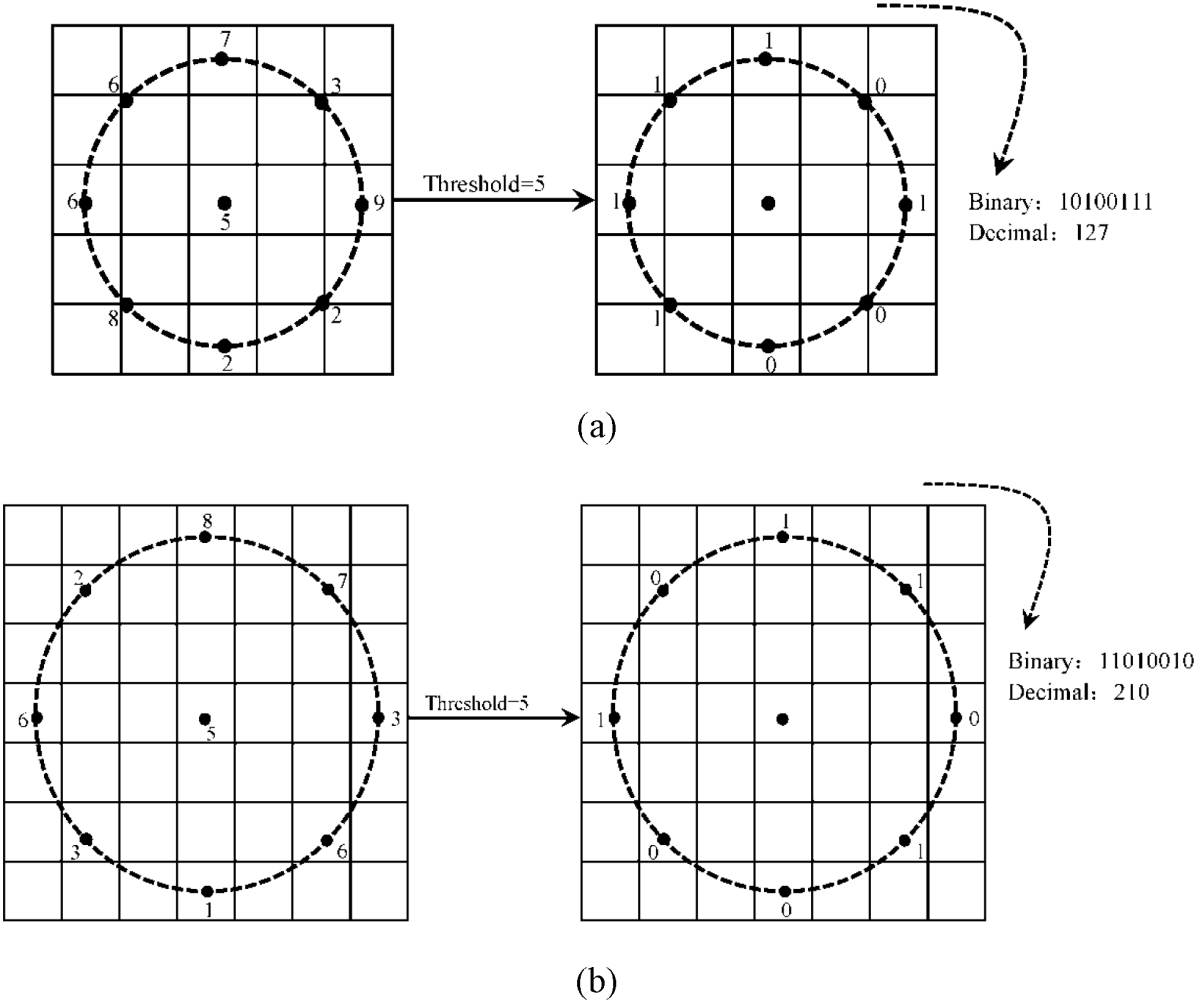
The examples of LBP using different neighborhood sizes. (a) d = 8,r = 5, (b) d = 8,r = 7.

Local Binary Pattern (LBP) [33] was original proposed by Ojala et al. as a powerful technique for texture description. It can efficiently describe the local texture of an image by thresholding each pixel in a 3 × 3 sized neighborhood with the center pixel's value and considering the results as a binary number (see Fig 1 for an illustration). As a result, 256-bin histogram of the LBP labels computed over the image can be used as a texture feature. To describe the image textures at different scales, the LBP was later extended to use different neighborhood sizes [33, 34]. In this way, the values of *d* points evenly sampled from a circle within an *r*×*r* sized neighborhood are compared with the center pixel’s value. Then, the comparison result can also be considered as a binary number (see Fig 2 for an illustration). When the sampled points are not exactly located in the centers of pixels, their values can be estimated by interpolation [33, 34].

Compared with other features, LBP feature has the advantage of invariant to monotone transformation. Thus, it is robust to the illumination and expression changes of face images to some extent and has been widely employed for face recognition. However, one limitation of LBP and its extensions is that the label information of face images is ignored. Therefore, the features extracted by these methods may lack of discrimination.

### Discriminant Face Descriptor

For the sake of overcoming the limitation of LBP and improving the discriminative ability of LBP features, Lei et al. proposed a discriminant image filter learning (IFL) for face recognition [19]. The main idea of IFL is to reduce the variances of LBP features of face images from intra person and meanwhile enlarge the margin between LBP features of face images from different persons. To achieve this goal, the label information of face images is utilized to learn a filter in IFL. Then, the LBP operator is applied on the filtered face images for local feature extraction. Specifically, let *I* denote an input face image and its filtered image is denoted as *f*(*I*). Considering the sampling strategy of LBP, IFL first defines pixel difference vector (PDV) as:

$$df{\left(I\right)}^{p}=[f{\left(I\right)}^{p_{1}}-f{\left(I\right)}^{p},f{\left(I\right)}^{p_{2}}-f{\left(I\right)}^{p},\cdots ,f{\left(I\right)}^{p_{d}}-f{\left(I\right)}^{p}],$$ (1)

where *f*(*I*)^*p*^ is the pixel value of filtered face image at position *p*, {*p*_1_, *p*_2_, ⋯, *p*_*d*_}∈*Neighbor*(*p*) and *d* is the number of sampled points. Then, in order to make sure that the PDVs of filtered face images from the same person are similar and the PDVs of filtered face images from different persons are distant. The Fisher criterion is adopted as

$$\text{max}\frac{S_{b}}{S_{w}},$$ (2)

where *S*_*b*_ and *S*_*w*_ are the between-class and within-class class scatters, which can be computed as

$$S_{b}=\sum_{i=1}^{L}C_{i}\left(df{\left(m\right)}_{i}-df\left(m\right)\right){\left(df{\left(m\right)}_{i}-df\left(m\right)\right)}^{T}$$ (3)

$$S_{w}=\sum_{i=1}^{L}\sum_{j=1}^{C_{i}}\left(df{\left(I\right)}_{ij}-df{\left(m\right)}_{i}\right){\left(df{\left(I\right)}_{ij}-df{\left(m\right)}_{i}\right)}^{T}$$ (4)

where *L* is the number of classes and *C*_*i*_ is the number of samples in the *i*-th class.$df{\left(I\right)}_{ij}=[df{\left(I\right)}_{ij}^{1},df{\left(I\right)}_{ij}^{2},\cdots ,df{\left(I\right)}_{ij}^{N}]$ is the PDV set from the *j*-th image of class *i*, and *N* is the number of PDV for each filtered face image. $df{\left(m\right)}_{i}=[df{\left(m\right)}_{i}^{1},df{\left(m\right)}_{i}^{2},\cdots ,df{\left(m\right)}_{i}^{N}]$ and *df*(*m*) = [*df*(*m*)^1^, *df*(*m*)^2^, ⋯, *df*(*m*)^*N*^] are augmented vectors by concatenating mean vectors over different positions.$df{\left(m\right)}_{i}^{p}$ is the mean vector of PDVs at position *p* of the filtered face images from the *i*-th class and *df*(*m*)^*P*^ is the total mean vector of PDVs at position *p* over the sample set. In IFL, the image filter vector is set to be *w*, and the value of filtered image at position *p* can be represented as *f*(*I*)^*P*^ = *w*^*T*^*I*^*p*^, where *I*^*p*^ denotes the patch vector centered at position *p* of the original face image. Therefore, the filter *w* can be learned by

$$\underset{w}{\text{max}}\frac{w^{T}{\hat{S}}_{b}w}{w^{T}{\hat{S}}_{w}w},$$ (5)

where ${\hat{S}}_{b}$ and ${\hat{S}}_{w}$ are the between-class and within-class scatters of PDVs from the original input face images. For more details about the IFL, the readers can refer to [19].

## The Proposed Algorithm

### Supervised Filter Learning

As shown in the previous section, IFL utilized the Fisher criterion to learn an optimal filter which improved the discriminative ability of LBP features extracted from filtered images. Therefore, like other methods based on Fisher criterion (such as LDA), it may only be suitable for the case in which the samples of each class are approximately Gaussian distributed [32]. However, this property is not always satisfied in face recognition problem [35]. Furthermore, the Fisher criterion is also not suitable for the representation based classification methods which have been proved to be effective for face recognition tasks. In order to overcome these limitations, we propose a new supervised filter learning (SFL) algorithm to improve the discriminative ability of LBP features for representation based face recognition.

Formally, let $T=\left[T_{1},T_{2},\cdots ,T_{N}\right]\in R^{D\times N}$ denote a set of training face images from *L* classes (each class possesses *C*_*i*_ samples, *i* = 1, …, *L*). Similar to IFL, we suppose that the filtered images are *f*(*T*) = [*f*(*T*_1_), *f*(*T*_2_), ⋯, *f*(*T*_*N*_)]. Since the proposed algorithm also applies LBP operator on the filtered image, we define the pixel difference vector (PDV) as:

$$df{\left(T_{i}\right)}^{p}={\left[f{\left(T_{i}\right)}^{p_{1}}-f{\left(T_{i}\right)}^{p},\cdots ,f{\left(T_{i}\right)}^{p_{d}}-f{\left(T_{i}\right)}^{p}\right]}^{T},\text{i}=1,\ldots ,\text{N}$$ (6)

where *f*(*T*_*i*_)^*p*^ is the pixel value of filtered face image *f*(*T*_*i*_) at position *p*, {*p*_1_, *p*_2_, ⋯, *p*_*d*_} ∈ *Neighbor*(*p*) and *d* is the number of sampled points.

Different from IFL which maximize the ratio of between-class scatter to the within-class scatter of LBP features extracted from the filtered face images, the aim of the proposed algorithm is to benefit the representation based face recognition methods. That is, our algorithm want to learn a filter so that after the image filtering, the LBP feature of a face image can be accurately represented by those from the same person and cannot be represented by those of different persons. To achieve this goal, we need to reduce the within-class representation residual and enlarge the between-class representation residual of the filtered images' PDVs. Suppose that *df*(*T*_*ij*_)^*p*^ is the *p*-th PDV of the *j*-th face image from the *i*-th class, its within-class representation residual can be obtained as:

$$r_{ij}^{w}={\left\|df{\left(T_{ij}\right)}^{p}-a_{ij}^{p}df{\left(T_{i\tilde{j}}\right)}^{p}\right\|}^{2}$$ (7)

where $df{\left(T_{i\tilde{j}}\right)}_{}^{p}\;=[df{\left(T_{i1}\right)}_{}^{p},\ldots df{\left(T_{ij-1}\right)}_{}^{p},\;df{\left(T_{ij+1}\right)}_{}^{p},\ldots df{\left(T_{iC_{i}}\right)}_{}^{p}]$ is a matrix formed by the *p*-th PDVs of the other filtered face images from the *i*-th class and $a_{ij}^{p}$ is the vector of within-class representation coefficients for *df*(*T*_*ij*_)^*p*^, which can be estimated using the least-squares algorithm [28] as:

$$a_{ij}^{p}={\left(df{\left(T_{i\tilde{j}}\right)}^{pT}\times df{\left(T_{i\tilde{j}}\right)}^{p}\right)}^{-1}df{\left(T_{i\tilde{j}}\right)}^{pT}df{\left(T_{ij}\right)}^{p}$$ (8)

where $df{\left(T_{i\tilde{j}}\right)}_{}^{pT}$ is the transpose of $df{\left(T_{i\tilde{j}}\right)}_{}^{p}$.

Considering all the PDVs, we can get the total within-class representation residual as

$$R^{w}={\sum_{i=1}^{L}\sum_{j=1}^{C_{i}}\sum_{p=1}^{D}\left\|df{\left(T_{ij}\right)}^{p}-a_{ij}^{p}df{\left(T_{i\tilde{j}}\right)}^{p}\right\|}^{2}$$ (9)

Similarly, the between-class representation residual of *df*(*T*_*ij*_)^*p*^ can be formulated as

$$r_{ij}^{b}={\left\|df{\left(T_{ij}\right)}^{p}-b_{ij}^{p}df{\left(T_{\tilde{i}•}\right)}^{p}\right\|}^{2}$$ (10)

where $df{\left(T_{\tilde{i}•}\right)}^{p}\;$ is a matrix formed by the *p*-th PDVs of the filtered face images do not belong to the *i*-th class and $b_{ij}^{p}$ is the vector of between-class representation coefficients for *df*(*T*_*ij*_)^*p*^, which can also be estimated by least-squares algorithm as:

$$b_{ij}^{p}={\left(df{\left(T_{\tilde{i}•}\right)}^{pT}\times df{\left(T_{\tilde{i}•}\right)}^{p}\right)}^{-1}df{\left(T_{\tilde{i}•}\right)}^{pT}df{\left(T_{ij}\right)}^{p}$$ (11)

where $df{\left(T_{\tilde{i}•}\right)}_{}^{pT}$ is the transpose of $df{\left(T_{\tilde{i}•}\right)}_{}^{p}$.

Then, the total between-class representation residual can be obtained as

$$R^{b}={\sum_{i=1}^{L}\sum_{j=1}^{C_{i}}\sum_{p=1}^{D}\left\|df{\left(T_{ij}\right)}^{p}-b_{ij}^{p}df{\left(T_{\tilde{i}•}\right)}^{p}\right\|}^{2}$$ (12)

Now, through combining Eqs (10) and (12), the objective function of our proposed filter learning algorithm is

$$\text{min}\frac{R^{w}}{R^{b}}$$ (13)

From the definitions of *R*^*w*^ and *R*^*b*^, it can be found that Eq (13) will incur heavy penalties if the within-class residual of the filtered images' PDVs is large and the between-class residual of the filtered images' PDVs is small. Thus, minimizing Eq (13) could ensure that the LBP features extracted from a filtered face image can only be well represented by those from the same class but cannot be represented by those from different classes. In this study, we suppose that the image filter with the size of *S* × *S* can be concatenated into a vector $\omega \in R^{F\times 1}$(*F* = *S* × *S*). Then, the value of a filtered image *f*(*T*_*ij*_) at position *p* can be denoted as $f{\left(T_{ij}\right)}^{p}=\omega^{T}T_{ij}^{p}$, where $T_{ij}^{p}$ is a vector concatenated by the patch centered at position *p* of image *T*_*ij*_. Analogously, the PDV at position *p* of a filtered image can also be denoted as $df{\left(T_{ij}\right)}^{p}=\omega^{T}dT_{ij}^{p}$, where $dT_{ij}^{p}$ is the PDV at position *p* of the unfiltered image *T*_*ij*_. Through substituting *df*(*T*_*ij*_)^*p*^ into Eqs (10) and (12), Eq (13) can be converted to

$$\begin{array}{l} \underset{\omega }{\text{min}}\frac{{\sum_{i=1}^{L}\sum_{j=1}^{C_{i}}\sum_{p=1}^{D}\left\|\omega^{T}dT_{ij}^{p}-a_{ij}^{p}\omega^{T}dT_{i\tilde{j}}^{p}\right\|}^{2}}{{\sum_{i=1}^{L}\sum_{j=1}^{C_{i}}\sum_{p=1}^{D}\left\|\omega^{T}dT_{ij}^{p}-b_{ij}^{p}\omega^{T}dT_{\tilde{i}•}^{p}\right\|}^{2}}\\ =\frac{\sum_{i=1}^{L}\sum_{j=1}^{C_{i}}\sum_{p=1}^{D}\omega^{T}(dT_{ij}^{p}-a_{ij}^{p}dT_{i\tilde{j}}^{p}){\left(dT_{ij}^{p}-a_{ij}^{p}dT_{i\tilde{j}}^{p}\right)}^{T}\omega }{\sum_{i=1}^{L}\sum_{j=1}^{C_{i}}\sum_{p=1}^{D}\omega^{T}(dT_{ij}^{p}-b_{ij}^{p}dT_{\tilde{i}•}^{p}){\left(dT_{ij}^{p}-b_{ij}^{p}dT_{\tilde{i}•}^{p}\right)}^{T}\omega } \end{array}$$ (14)

Let ${\hat{R}}^{w}=\sum_{i=1}^{L}\sum_{j=1}^{C_{i}}\sum_{p=1}^{D}\left(dT_{ij}^{p}-a_{ij}^{p}dT_{i\tilde{j}}^{p}\right){\left(dT_{ij}^{p}-a_{ij}^{p}dT_{i\tilde{j}}^{p}\right)}^{T}$ and ${\hat{R}}^{b}=\sum_{i=1}^{L}\sum_{j=1}^{C_{i}}\sum_{p=1}^{D}(dT_{ij}^{p}-b_{ij}^{p}dT_{\tilde{i}•}^{p}){\left(dT_{ij}^{p}-b_{ij}^{p}dT_{\tilde{i}•}^{p}\right)}^{T}$, Eq (14) is reduced to

$$\underset{\omega }{\text{min}}\frac{\omega^{T}{\hat{R}}^{w}\omega }{\omega^{T}{\hat{R}}^{b}\omega }$$ (15)

From Eq (15), it is clear that both the matrix ${\hat{R}}^{w}$ and ${\hat{R}}^{b}$ are symmetric and positive semi-definite. As a result, the optimal filter (i.e. *ω*) that minimizing the objective function of our algorithm can be obtained by solving the generalized eigenvalue problem ${\hat{R}}^{w}\omega =\lambda {\hat{R}}^{b}\omega$ with its smallest eigenvalue.

After the filter *ω* has been learned, we can convert it into the matrix form with the size of *S* × *S* and employ it to filter the training face images. Then, the LBP features are extracted from the filtered images and the representation based classification methods (such as SRC and LSR) can be utilized for recognition.

### Extended SFL for Heterogeneous Face Recognition

Nowadays, heterogeneous face image recognition has attracted more and more attentions due to its widely applications in video surveillance and law enforcement. According to some studies [19, 36], the heterogeneous faces can be defined as faces which are captured in different environments or different devices. For instance, the face images captured by visible light and near-infrared imaging devices can be regarded as heterogeneous faces.

In this section, we extended the proposed SFL for heterogeneous face recognition problem. Similar to the SFL for homogeneous face images, the aim of extended SFL is to learn a filter to reduce the within-class representation residual of faces’ LBP features for heterogeneous images from the same person and enlarge the between-class representation residual of faces' LBP features for heterogeneous images from the different persons. Suppose $T^{V}=\left[T_{1}^{V},T_{2}^{V},\cdots ,T_{N}^{V}\right]\in R^{D\times N}$ and $T^{M}=\left[T_{1}^{M},T_{2}^{M},\cdots ,T_{N}^{M}\right]\in R^{D\times N}$ are two heterogeneous image sets (e.g. images captured by visible light and near-infrared imaging devices), and the filtered images of them are $f(T^{V})=\left[f(T_{1}^{V}),f(T_{2}^{V}),\cdots ,f(T_{N}^{V})\right]\in R^{D\times N}$ and $f(T^{M})=\left[f(T_{1}^{M}),f(T_{2}^{M}),\cdots ,f(T_{N}^{M})\right]\in R^{D\times N}$, respectively. Let $df{\left(T_{ij}^{V}\right)}_{}^{p}$ and $df{\left(T_{ij}^{M}\right)}_{}^{p}$ be the *p*-th PDVs of the *j*-th faces from the *i*-th class in two heterogeneous image sets. In order to make sure that the LBP features of face images can be well represented by those from the same person, we need to minimize the following within-class representation residual:

$${\tilde{R}}^{w}=R_{VV}^{w}+R_{VM}^{w}+R_{MV}^{w}+R_{MM}^{w}$$ (16)

where $R_{VV}^{w}$ and $R_{MM}^{w}$ are the homogeneous within-class representation residuals which can be obtained by Eq (9). $R_{VM}^{w}$ and $R_{MV}^{w}$ are the within-class representation residuals between heterogeneous images, which can be defined as:

$$R_{{}^{VM}}^{w}={\sum_{i=1}^{L}\sum_{j=1}^{C_{i}}\sum_{p=1}^{D}\left\|df{\left(T_{ij}^{V}\right)}^{p}-a_{ij}^{VM}{}^{p}df{\left(T_{i•}^{M}\right)}^{p}\right\|}^{2}$$ (17)

and

$$R_{{}^{MV}}^{w}={\sum_{i=1}^{L}\sum_{j=1}^{C_{i}}\sum_{p=1}^{D}\left\|df{\left(T_{ij}^{M}\right)}^{p}-a_{ij}^{MV}{}^{p}df{\left(T_{i•}^{V}\right)}^{p}\right\|}^{2}$$ (18)

where $df{\left(T_{i•}^{M}\right)}^{p}$ and $df{\left(T_{i•}^{V}\right)}^{p}$ are the matrices formed by the *p*-th PDVs of face images from the *i*-th class in image sets *T*^*M*^ and *T*^*V*^. $a_{ij}^{VM}{}^{p}$ and $a_{ij}^{MV}{}^{p}$ are the heterogeneous representation coefficients of $df{\left(T_{ij}^{V}\right)}^{p}$ and $df{\left(T_{ij}^{M}\right)}^{p}$, which can be obtained by least-squares algorithm similar to Eq (8).

Analogically, to ensure that the LBP features of face images cannot be represented by those from different persons, the following between-class representation residual should be maximized:

$${\tilde{R}}^{b}=R_{VV}^{b}+R_{VM}^{b}+R_{MV}^{b}+R_{MM}^{b}$$ (19)

where $R_{VV}^{b}$ and $R_{MM}^{b}$ are the homogeneous between-class representation residuals obtained by Eq (12). $R_{VM}^{b}$ and $R_{MV}^{b}$ are the heterogeneous between-class representation residuals defined as:

$$R_{VM}^{b}={\sum_{i=1}^{L}\sum_{j=1}^{C_{i}}\sum_{p=1}^{D}\left\|df{\left(T_{ij}^{V}\right)}^{p}-b_{ij}^{VM}{}^{p}df{\left(T_{\tilde{i}•}^{M}\right)}^{p}\right\|}^{2}$$ (20)

and

$$R_{{}^{MV}}^{b}={\sum_{i=1}^{L}\sum_{j=1}^{C_{i}}\sum_{p=1}^{D}\left\|df{\left(T_{ij}^{M}\right)}^{p}-b_{ij}^{MV}{}^{p}df{\left(T_{\tilde{i}•}^{V}\right)}^{p}\right\|}^{2}$$ (21)

where $df{\left(T_{\tilde{i}•}^{M}\right)}^{p}$ and $df{\left(T_{\tilde{i}•}^{V}\right)}^{p}$ are the matrices formed by the *p*-th PDVs of face images do not belong to the *i*-th class in image sets *T*^*M*^ and *T*^*V*^. $b_{ij}^{VM}{}^{p}$ and $b_{ij}^{MV}{}^{p}$ are the heterogeneous representation coefficients of $df{\left(T_{ij}^{V}\right)}^{p}$ and $df{\left(T_{ij}^{M}\right)}^{p}$.

Through combining Eqs (16) and (19) together, we can obtain the objective function of extended SFL for heterogeneous face recognition as

$$\text{min}\frac{{\tilde{R}}^{w}}{{\tilde{R}}^{b}}$$ (22)

Similar to ‘Supervised Filter Learning’ section, we also suppose that that the image filter with the size of *S × S* can be concatenated into a vector $\omega \in R^{F\times 1}$(*F* = *S × S*). Then, we have $df{\left(T_{ij}^{M}\right)}^{p}=\omega^{T}dT_{ij}^{M}{}^{p}$ and $df{\left(T_{ij}^{V}\right)}^{p}=\omega^{T}dT_{ij}^{V}{}^{p}$, where $dT_{ij}^{M}{}^{p}$ and $dT_{ij}^{M}{}^{p}$ are the PDVs at position *p* of the unfiltered images $T_{ij}^{M}$ and $T_{ij}^{V}$. Now, by substituting $df{\left(T_{ij}^{V}\right)}^{p}$ and $df{\left(T_{ij}^{M}\right)}^{p}$ into Eqs (16) and (19), these two equations can be converted to

$$\begin{array}{l} {\tilde{R}}^{w}=R_{VV}^{w}+R_{VM}^{w}+R_{MV}^{w}+R_{MM}^{w}\\ ={\sum_{i=1}^{L}\sum_{j=1}^{C_{i}}\sum_{p=1}^{D}\left\|\omega^{T}dT_{ij}^{V}{}^{p}-a_{ij}^{VV}{}^{p}\omega^{T}dT_{i\tilde{j}}^{V}{}^{p}\right\|}^{2}+{\sum_{i=1}^{L}\sum_{j=1}^{C_{i}}\sum_{p=1}^{D}\left\|\omega^{T}dT_{ij}^{V}{}^{p}-a_{ij}^{VM}{}^{p}\omega^{T}dT_{i•}^{M}{}^{p}\right\|}^{2}\\ +{\sum_{i=1}^{L}\sum_{j=1}^{C_{i}}\sum_{p=1}^{D}\left\|\omega^{T}dT_{ij}^{M}{}^{p}-a_{ij}^{MV}{}^{p}\omega^{T}dT_{i•}^{V}{}^{p}\right\|}^{2}+\sum_{i=1}^{L}\sum_{j=1}^{C_{i}}\sum_{p=1}^{D}\left\|\omega^{T}dT_{ij}^{M}{}^{p}-a_{ij}^{MM}{}^{p}\omega^{T}dT_{i\tilde{j}}^{M}{}^{p}\right\| \end{array}$$ (23)

$$\begin{array}{l} {\tilde{R}}^{b}=R_{VV}^{b}+R_{VM}^{b}+R_{MV}^{b}+R_{MM}^{b}\\ ={\sum_{i=1}^{L}\sum_{j=1}^{C_{i}}\sum_{p=1}^{D}\left\|\omega^{T}dT_{ij}^{V}{}^{p}-b_{ij}^{VV}{}^{p}\omega^{T}dT_{\tilde{i}•}^{V}{}^{p}\right\|}^{2}+{\sum_{i=1}^{L}\sum_{j=1}^{C_{i}}\sum_{p=1}^{D}\left\|\omega^{T}dT_{ij}^{V}{}^{p}-b_{ij}^{VM}{}^{p}\omega^{T}dT_{\tilde{i}•}^{M}{}^{p}\right\|}^{2}\\ +\sum_{i=1}^{L}\sum_{j=1}^{C_{i}}\sum_{p=1}^{D}{\left\|\omega^{T}dT_{ij}^{M}{}^{p}-b_{ij}^{MV}{}^{p}\omega^{T}dT_{\tilde{i}•}^{V}{}^{p}\right\|}^{2}+{\sum_{i=1}^{L}\sum_{j=1}^{C_{i}}\sum_{p=1}^{D}\left\|\omega^{T}dT_{ij}^{M}{}^{p}-b_{ij}^{MM}{}^{p}\omega^{T}dT_{\tilde{i}•}^{M}{}^{p}\right\|}^{2} \end{array}$$ (24)

After a series of deductions, Eq (22) can be reduced to

$$\text{min}\frac{\omega^{T}K^{w}\omega }{\omega^{T}K^{b}\omega }$$ (25)

where

$$\begin{array}{l} K^{w}={\sum_{i=1}^{L}\sum_{j=1}^{C_{i}}\sum_{p=1}^{D}\left(\omega^{T}dT_{ij}^{V}{}^{p}-a_{ij}^{VV}{}^{p}\omega^{T}dT_{i\tilde{j}}^{V}{}^{p}\right)\left(\omega^{T}dT_{ij}^{V}{}^{p}-a_{ij}^{VV}{}^{p}\omega^{T}dT_{i\tilde{j}}^{V}{}^{p}\right)}^{T}\\ +{\sum_{i=1}^{L}\sum_{j=1}^{C_{i}}\sum_{p=1}^{D}\left(\omega^{T}dT_{ij}^{V}{}^{p}-a_{ij}^{VM}{}^{p}\omega^{T}dT_{i•}^{M}{}^{p}\right)\left(\omega^{T}dT_{ij}^{V}{}^{p}-a_{ij}^{VM}{}^{p}\omega^{T}dT_{i•}^{M}{}^{p}\right)}^{T}\\ +{\sum_{i=1}^{L}\sum_{j=1}^{C_{i}}\sum_{p=1}^{D}\left(\omega^{T}dT_{ij}^{M}{}^{p}-a_{ij}^{MV}{}^{p}\omega^{T}dT_{i•}^{V}{}^{p}\right)\left(\omega^{T}dT_{ij}^{M}{}^{p}-a_{ij}^{MV}{}^{p}\omega^{T}dT_{i•}^{V}{}^{p}\right)}^{T}\\ +{\sum_{i=1}^{L}\sum_{j=1}^{C_{i}}\sum_{p=1}^{D}\left(\omega^{T}dT_{ij}^{M}{}^{p}-a_{ij}^{MM}{}^{p}\omega^{T}dT_{i\tilde{j}}^{M}{}^{p}\right)\left(\omega^{T}dT_{ij}^{M}{}^{p}-a_{ij}^{MM}{}^{p}\omega^{T}dT_{i\tilde{j}}^{M}{}^{p}\right)}^{T} \end{array}$$ (26)

$$\begin{array}{l} K^{b}=\sum_{i=1}^{L}\sum_{j=1}^{C_{i}}\sum_{p=1}^{D}\left(\omega^{T}dT_{ij}^{V}{}^{p}-b_{ij}^{VV}{}^{p}\omega^{T}dT_{\tilde{i}•}^{V}{}^{p}\right){\left(\omega^{T}dT_{ij}^{V}{}^{p}-b_{ij}^{VV}{}^{p}\omega^{T}dT_{\tilde{i}•}^{V}{}^{p}\right)}^{T}\\ +\sum_{i=1}^{L}\sum_{j=1}^{C_{i}}\sum_{p=1}^{D}\left(\omega^{T}dT_{ij}^{V}{}^{p}-b_{ij}^{VM}{}^{p}\omega^{T}dT_{\tilde{i}•}^{M}{}^{p}\right){\left(\omega^{T}dT_{ij}^{V}{}^{p}-b_{ij}^{VM}{}^{p}\omega^{T}dT_{\tilde{i}•}^{M}{}^{p}\right)}^{T}\\ +\sum_{i=1}^{L}\sum_{j=1}^{C_{i}}\sum_{p=1}^{D}\left(\omega^{T}dT_{ij}^{M}{}^{p}-b_{ij}^{MV}{}^{p}\omega^{T}dT_{\tilde{i}•}^{V}{}^{p}\right){\left(\omega^{T}dT_{ij}^{M}{}^{p}-b_{ij}^{MV}{}^{p}\omega^{T}dT_{\tilde{i}•}^{V}{}^{p}\right)}^{T}\\ +\sum_{i=1}^{L}\sum_{j=1}^{C_{i}}\sum_{p=1}^{D}\left(\omega^{T}dT_{ij}^{M}{}^{p}-b_{ij}^{MM}{}^{p}\omega^{T}dT_{\tilde{i}•}^{V}{}^{p}\right){\left(\omega^{T}dT_{ij}^{M}{}^{p}-b_{ij}^{MM}{}^{p}\omega^{T}dT_{\tilde{i}•}^{M}{}^{p}\right)}^{T} \end{array}$$ (27)

Therefore, the optimal filter *ω* that minimizing the objective function of extended SFL in Eq (25) can be obtained by solving the generalized eigenvalue problem *K*^*w*^*ω* = *λK*^*b*^*ω* with its smallest eigenvalue. After the filter learning, *ω* can be converted into its matrix form to filter the heterogeneous face images in *T*^*M*^ and *T*^*V*^. Then, the SRC or LRC can be adopted for recognition.

## Experiments

In this section, the performance of the proposed algorithm is tested and compared with other related algorithms such as LBP [33], LGBP [24], GVLBP [25], IFL-LBP [19], DSNPE [37], MNSMC [38]and UDSPP [39]. Among these algorithms, LBP, LGBP, GVLBP and IFL-LBP are LBP based methods, while DSNPE, MNSMC and UDSPP are recently proposed subspace based methods for representation based face recognition. Here, five benchmark face databases including Yale [40], AR [41], CMU PIE [42], LFW [43] and VLNHF [44] are employed. The proposed algorithm and other approaches used for comparison are all implemented in Matlab and executed on a computer with Intel Core i3-2100 CPU at 3.2 GHz and 8 GB physical memory.

### Data Description

The Yale face database [40] contains 165 grayscale images of 15 individuals. Thereare11 images per subject, and the images demonstrate variations in facial expression (normal, sad, happy, sleepy, surprised, and wink), lighting condition (left-light, center-light, right-light), and with/without glasses. In our experiment, 6 images of each person are randomly selected for training and the rest images are used for testing.

The AR face database [41] consists of more than 4000 frontal images from 126 subjects including 70 males and 56 females. The images were taken in two sessions separated by two weeks with expression (neutral, smile, anger and scream) and occlusion (sunglass and scarf) variations. In this experiment, we choose a subset which contains 50 males and 50 females. For each subject, 14 images with only illumination and expression changes are selected. We randomly select 7 images from each person for training, and remaining images are used for testing.

The CMU PIE face database [42] includes 68 subjects with 41368 face images as a whole, each subject contains 13 different poses, 43 different illumination conditions, and 4 different expressions. In our experiment, 24 face images of each individual are used. For this database, we randomly select 12 images of each person to form the training set and the rest images are utilized for testing.

The LFW database [43] is a large scale database which contains 13,233 face images of 5,749 different individuals. Since all the samples were taken from the real world in an unconstrained environment, the expression, pose, illumination, occlusions and alignment of face images are very variable in this database. In our study, a subset which contains 1580 face images of 158 individuals from the LFW database is employed. We randomly select 7 images from each person for training, and remaining images are used for testing.

The Visible Light and Near-infrared Human Face (VLNHF) database [44] is a heterogeneous face image database which consists of two datasets (Lab1 and Lab2). The Lab1 dataset simultaneously contains visible light images and near-infrared images of 50 persons. Each person has 10 visible light images and 10 near-infrared images. The Lab2 dataset also contains visible light images and near-infrared images of 50 subjects. Each subject provides twenty visible light face images and the same number of near-infrared face images. These images were acquired under four different illumination conditions, and also have variation in facial expression and pose. In the experiment, 7 visible light images and 7 near-infrared images of each person are randomly selected for training in Lab1 dataset, and 12 visible light images and 12 near-infrared images of each person are randomly selected for training in Lab2 dataset. The rest images are used for testing.

In our recognition experiment, all images are manually aligned, cropped, and then resized to the resolution of 66×66, the random training sample selection are repeated 10 times for all databases and the averaged recognition accuracies are reported in the next subsection.

### Results and Discussions

In the proposed algorithm and IFL-LBP, the image filter size *S* and neighborhood size *r* of LBP will affect their performances. According to [36], we empirically set *S* and *r* to be the same value and tune the value from{3, 5, 7}. The number of sampled points is set as *d* = 8 for all LBP based algorithms so that 256 dimension LBP features are extracted. For DSNPE, MNSMC and UDSPP, in order to fairly compare them with the LBP based algorithms, the dimension of subspace in these three algorithms are also set as 256. Two well known representation based classifiers, i.e., SRC [2] and LRC [3] are adopted for recognition in our study.

#### Homogeneous face recognition

The recognition performances of various approaches on different homogeneous face databases can be seen in Tables 1–4. From these tables, the following points can be observed. Firstly, it can be found that LBP extracts the local texture features directly from the original face images, so its performances are inferior to other algorithms in most cases. Secondly, we can see that the performances of LGBP and GVLBP are better than LBP on AR, CMU PIE and LFW databases. This is because that LGBP and GVLBP extract the LBP features from the images after multi-scale and multi-orientation Gabor filtering, which could eliminate the influences of illumination and expression changes in the face images to some extent. However, we can also observe that LBP outperforms the LGBP and GVLBP on Yale database. The reason to this phenomenon may be that the number of individuals in Yale is much less than other three databases. Thus, the dimension of LBP features obtained from multi-scale and multi-orientation Gabor filtered face images is much higher than the number of training instances. This “small sample size” problem will weaken the performances of classifiers [45]. Thirdly, since IFL-LBP learns the filter in a supervised manner, its recognition results are better than other LBP based algorithms. Fourthly, we can find that the performances of subspace based algorithms (i.e. DSNPE, MNSMC and UDSPP) are better than LBP, LGBP, GVLBP and IFL-LBP in some cases. This is because these three algorithms are all designed for representation based face recognition. Nevertheless, since the subspace based algorithms only extract holistic features from the face images, their recognition results are still worse than our algorithm. At last, it can be seen that the proposed algorithm outperforms IFL-LBP and other algorithms on all databases. This is due to that the filter in our algorithm is learned based on the representation residual rather than Fisher criterion, which makes the LBP features extracted from the filtered images more suitable for the representation based classifiers. Besides the representation based classifiers, we also compare the performances of our SFL-LBP with IFL-LBP using Nearest Neighbor classifier. From the experimental results in Table 5, it can be found that the proposed algorithm outperforms IFL-LBP in most cases. This is because the Fisher criterion utilized in IFL cannot work well when the input training samples are not Gaussian distributed.

**Table 1:** The average recognition rates (%) and standard deviations (%) of different algorithms on Yale database.

Algorithms	SRC	LRC
LBP	92.53±1.68	93.20±3.29
LGBP	92.93±1.78	91.20±2.52
GVLBP	91.73±2.49	92.00±3.77
DSNPE	92.53±2.82	82.80±2.47
MNSMC	93.46±2.03	82.53±2.21
UDSPP	94.13±1.56	82.13±4.36
IFL-LBP(*S = r* = 3)	93.07±2.15	94.13±3.02
IFL-LBP(*S = r* = 5)	92.40±2.18	89.87±3.93
IFL-LBP(*S = r* = 7)	89.57±2.97	88.93±2.88
SFL-LBP(*S = r* = 3)	96.40±2.74	96.40±1.89
SFL-LBP(*S = r* = 5)	95.87±2.47	93.87±2.74
SFL-LBP(*S = r* = 7)	92.80±2.60	93.87±2.96

**Table 2:** Theaverage recognition rates (%) and standard deviations (%) of different algorithms on AR database.

Algorithms	SRC	LRC
LBP	89.40±1.33	81.99±1.50
LGBP	90.89±0.96	87.99±1.25
GVLBP	91.17±1.15	90.41±1.07
DSNPE	90.91±1.05	87.38±1.44
MNSMC	91.26±1.58	86.05±1.22
UDSPP	90.72±1.27	90.15±1.81
IFL-LBP(*S = r* = 3)	90.24±0.92	90.90±1.08
IFL-LBP(*S = r* = 5)	92.80±1.35	89.94±2.10
IFL-LBP(*S = r* = 7)	91.63±1.81	88.76±2.06
SFL-LBP(*S = r* = 3)	92.13±0.94	91.94±1.04
SFL-LBP(*S = r* = 5)	93.93±0.75	91.59±1.25
SFL-LBP(*S = r* = 7)	93.20±1.06	90.77±1.08

**Table 3:** Theaverage recognition rates (%) and standard deviations (%) of different algorithms on CMUPIE database.

Algorithms	SRC	LRC
LBP	91.67±0.40	88.32±0.85
LGBP	90.31±0.87	89.31±1.23
GVLBP	91.86±0.91	90.75±0.72
DSNPE	91.74±0.41	91.65±0.84
MNSMC	91.58±0.96	91.08±0.81
UDSPP	89.40±0.76	91.46±0.80
IFL-LBP(*S = r* = 3)	91.91±0.62	91.47±0.53
IFL-LBP(*S = r* = 5)	92.45±1.06	90.92±0.66
IFL-LBP(*S = r* = 7)	92.82±0.95	91.72±0.72
SFL-LBP(*S = r* = 3)	92.23±0.59	92.17±1.09
SFL-LBP(*S = r* = 5)	92.79±0.76	91.47±0.49
SFL-LBP(*S = r* = 7)	93.24±0.63	92.14±0.99

**Table 4:** The average recognition rates (%) and standard deviations (%) of different algorithms on LFW database.

Algorithms	SRC	LRC
LBP	34.21±1.17	42.25±1.10
LGBP	35.06±1.34	42.82±1.83
GVLBP	38.23±1.81	42.73±1.29
DSNPE	34.02±1.48	40.07±1.37
MNSMC	36.89±1.40	42.97±2.80
UDSPP	36.77±2.25	42.60±1.76
IFL-LBP(*S* = *r* = 3)	37.81±1.63	43.29±2.24
IFL-LBP(*S* = *r* = 5)	40.21±2.03	42.07±2.21
IFL-LBP(*S* = *r* = 7)	38.77±2.10	43.21±1.90
SFL-LBP(*S* = *r* = 3)	42.55±1.55	47.41±2.18
SFL-LBP(*S* = *r* = 5)	42.05±2.73	46.54±2.96
SFL-LBP(*S* = *r* = 7)	41.87±1.26	46.28±2.74

**Table 5:** The average recognition rates (%) and standard deviations (%) obtained by IFL-LBP and SFL-LBP using Nearest Neighbor classifier.

Algorithms	Yale	AR	CMU PIE	LFW
IFL-LBP(*S = r* = 3)	95.87±2.62	90.71±0.76	92.79±0.74	34.05±1.08
IFL-LBP(*S = r* = 5)	94.00±2.10	89.90±1.33	92.11±0.53	36.79±1.79
IFL-LBP(*S = r* = 7)	91.07±3.66	85.73±0.74	92.29±0.41	34.79±1.53
SFL-LBP(*S = r* = 3)	96.00±2.67	89.17±0.81	92.87±0.61	38.02±2.20
SFL-LBP(*S = r* = 5)	92.80±3.72	90.26±0.41	92.60±0.49	37.11±2.91
SFL-LBP(*S = r* = 7)	90.80±3.99	88.43±1.44	92.88±0.54	37.53±1.81

Then, the performances of our algorithm under different filter and neighborhood sizes are compared. From the experimental results in Tables 1–4, it can be found that the proposed SFL-LBP achieves better performances than IFL-LBP when their parameters are set as the same value. Moreover, we can also see that the values of parameters *S* and *r* have important effect on the performances of both IFL-LBP and SFL-LBP. However, given the standard deviation, the differences among the recognition results of our algorithm under various parameter values are less than IFL-LBP (especially on AR and CMU PIE databases). This indicates the proposed algorithm is less sensitive to the parameters when they are set as appropriate values.

**Fig 3:**
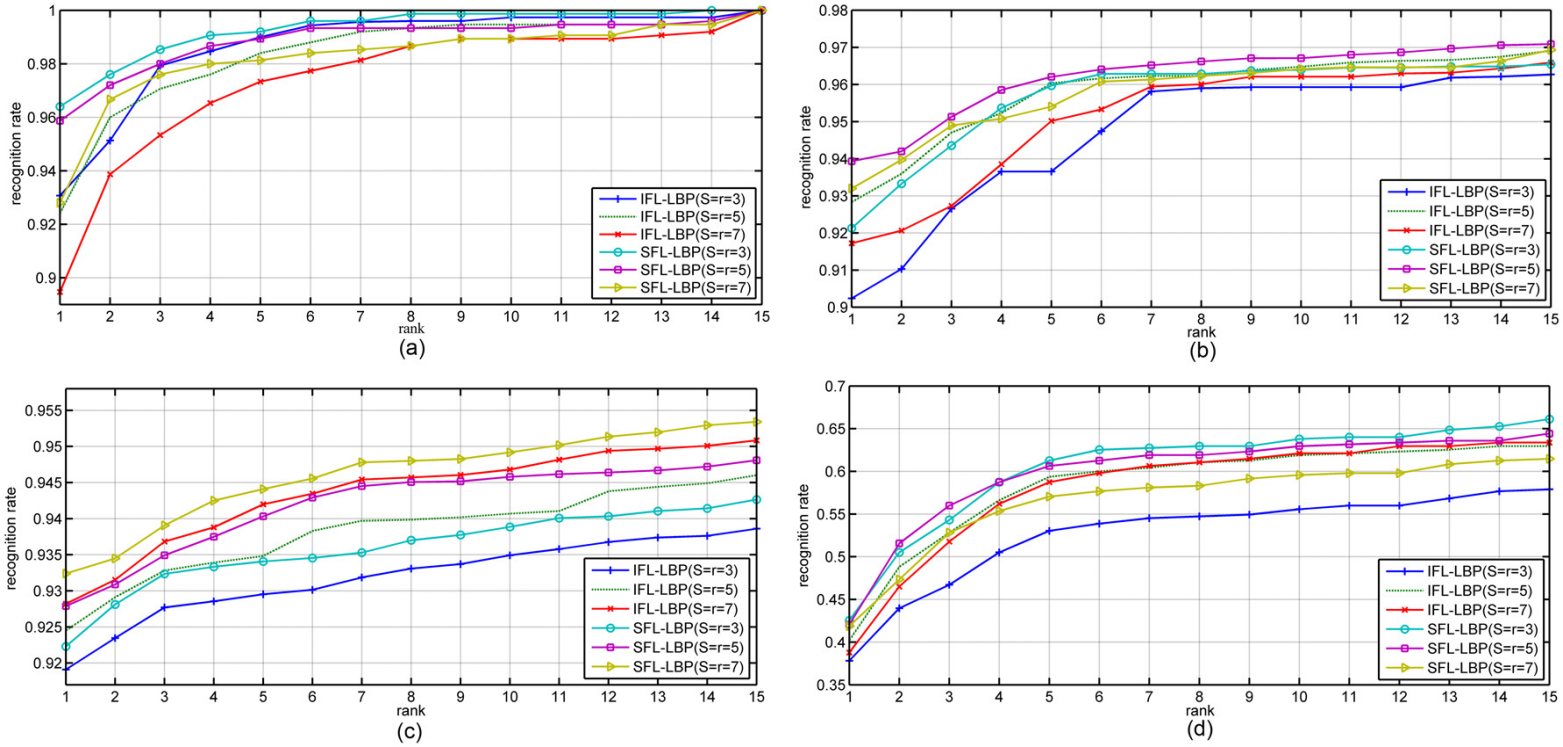
The CMC curves obtained by IFL-LBP and our SFL-LBP on different databases using SRC as classifier (a) Yale, (b) AR, (c) CMU PIE and (d) LFW.

**Fig 4:**
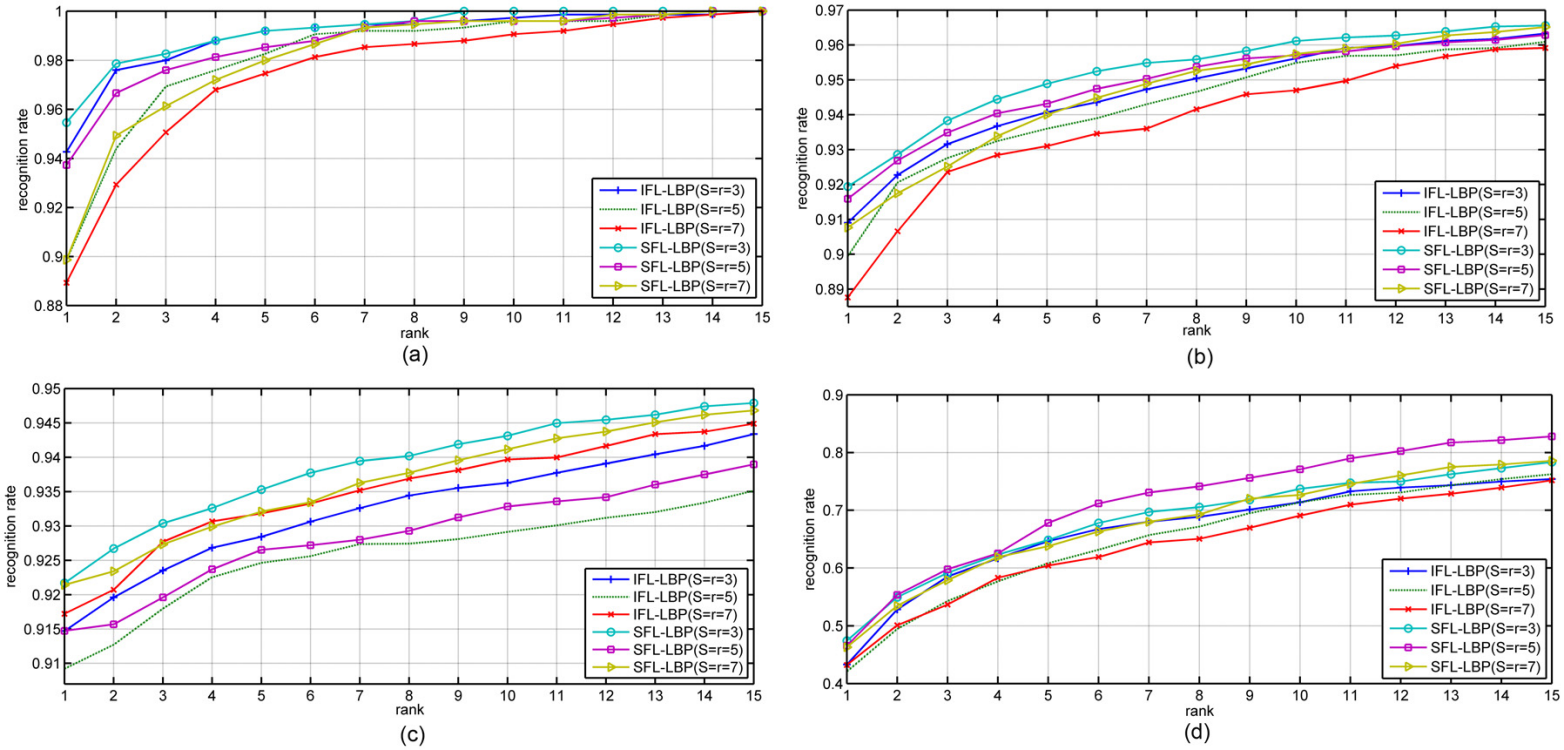
The CMC curves obtained by IFL-LBP and our SFL-LBP on different databases using LRC as classifier (a) Yale, (b) AR, (c) CMU PIE and (d) LFW.

Next, the Cumulative Match Characteristic (CMC) curve is used in our experiment to further compare the performances of IFL-LBP and our algorithm. From the CMC curves in Figs 3 and 4, it can be observed that our algorithm outperforms IFL-LBP nearly at all ranks, which demonstrates the advantage and robust of our algorithm for representation based face recognition tasks.

#### Heterogeneous face recognition

**Fig 5:**
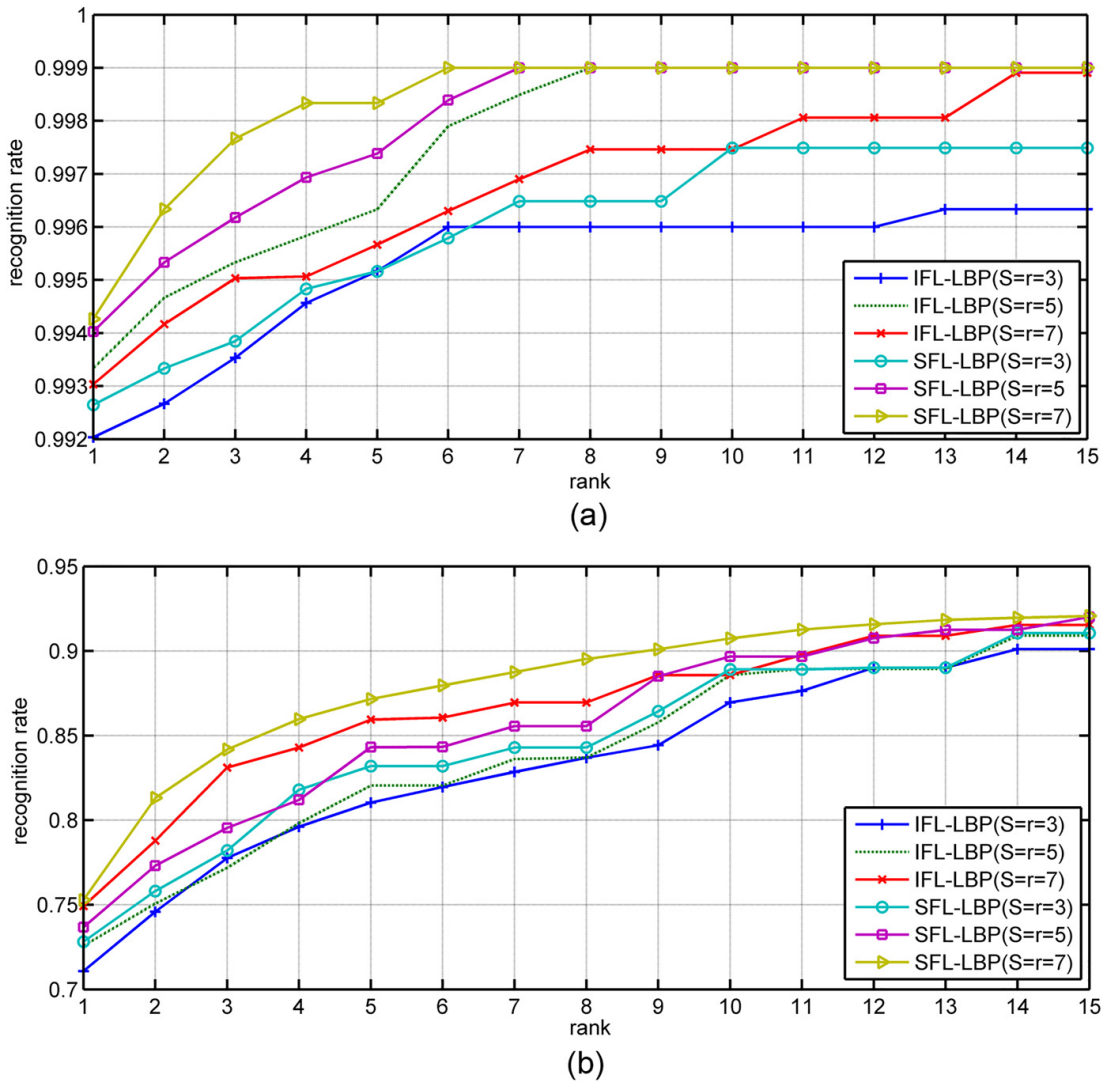
The CMC curves obtained by IFL-LBP and our SFL-LBP on VLNHF database using SRC as classifier (a) Lab1 dataset, (b) Lab2 dataset.

**Fig 6:**
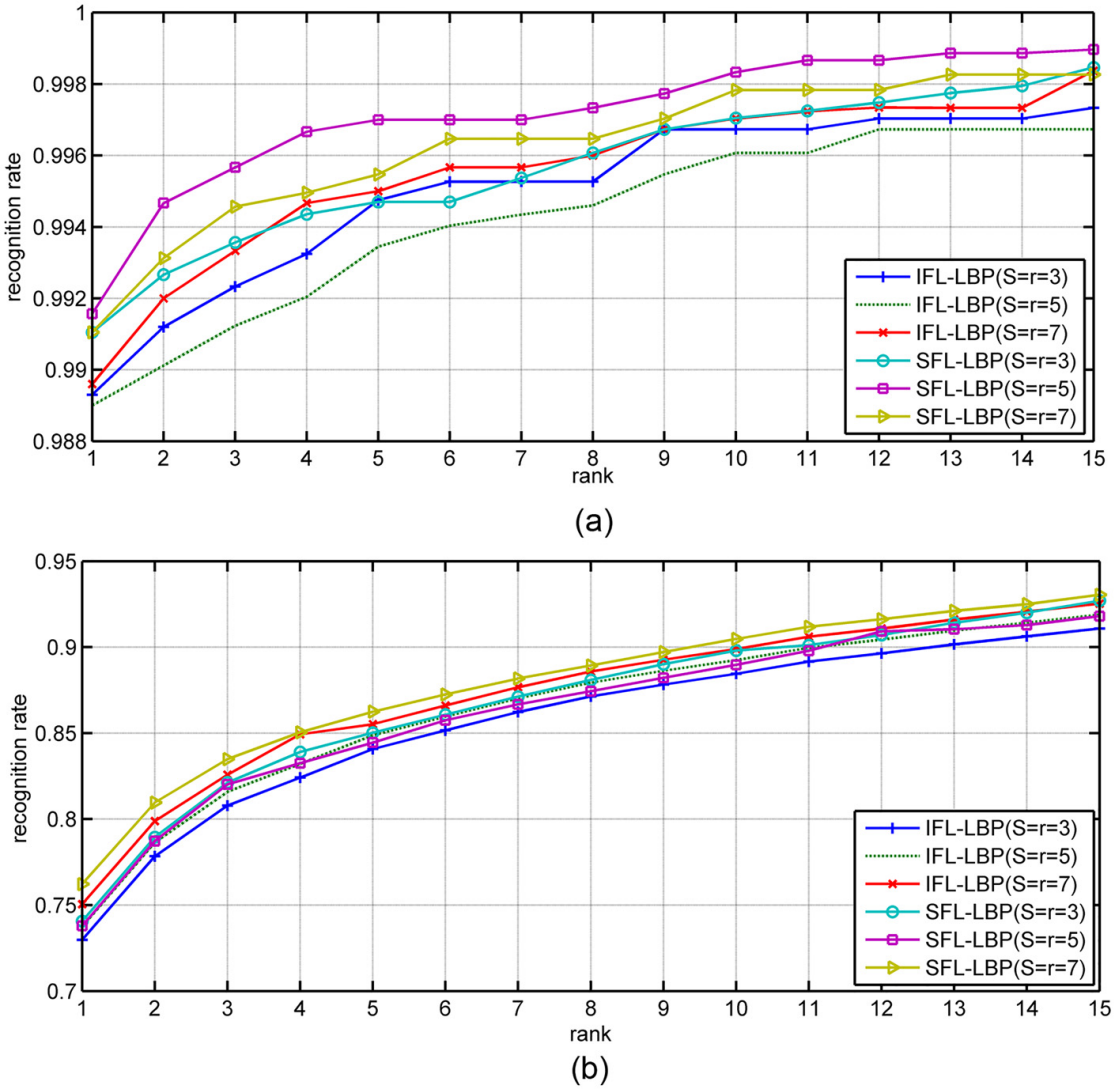
The CMC curves obtained by IFL-LBP and our SFL-LBP on VLNHF database using LRC as classifier (a) Lab1 dataset, (b) Lab2 dataset.

In this subsection, the performance of the proposed algorithm for heterogeneous face recognition are validated and compared with IFL. The average recognition results obtained by IFL and SFL are tabulated in Tables 6 and 7. From these tables, we can find that the proposed SFL outperforms IFL, which is consistent with the experimental results in ‘Homogeneous Face Recognition’ section. Furthermore, from CMC curves in Figs 5 and 6, the superior of our SFL for heterogeneous face recognition task is also verified.

**Table 6:** The average recognition rates (%) and standard deviations (%) of IFL-LBP and SFL-LBP algorithms on Lab1 of VLNHF database.

Algorithms	SRC	LRC
IFL-LBP(*S* = *r* = 3)	99.20±0.52	98.93±0.64
IFL-LBP(*S* = *r* = 5)	99.33±0.47	98.90±0.66
IFL-LBP(*S* = *r* = 7)	99.30±0.48	98.96±0.72
SFL-LBP(*S* = *r* = 3)	99.26±0.43	99.10±0.64
SFL-LBP(*S* = *r* = 5)	99.40±1.16	99.16±0.47
SFL-LBP(*S* = *r* = 7)	99.43±0.31	99.10±0.49

**Table 7:** The average recognition rates (%) and standard deviations (%) of IFL-LBP and SFL-LBP algorithms on Lab2 of VLNHF database.

Algorithms	SRC	LRC
IFL-LBP(*S* = *r* = 3)	71.08±2.05	72.98±2.18
IFL-LBP(*S* = *r* = 5)	72.57±3.50	73.75±3.48
IFL-LBP(*S* = *r* = 7)	74.92±3.27	75.05±2.97
SFL-LBP(*S* = *r* = 3)	72.82±2.25	74.06±1.39
SFL-LBP(*S* = *r* = 5)	73.07±1.88	73.78±1.74
SFL-LBP(*S* = *r* = 7)	75.28±1.94	76.22±2.24

#### Statistical test

In this subsection, the one-tailed Wilcoxon rank sum test is utilized to verify whether the performance of our algorithm is significantly better than the other algorithms. In this test, the null hypothesis is that the proposed SFL-LBP makes no difference when compared to other algorithms, and the alternative hypothesis is that SFL-LBP makes an improvement when compared to other algorithms. For instance, if we want to compare the performance of our algorithm with that of LBP (SFL-LBP vs. LBP), the null and alternative hypotheses can be defined as *H*_0_: M_SFL-LBP_ = M_LBP_ and *H*_1_: M_SFL-LBP_> M_LBP_, where M_SFL-LBP_ and M_LBP_ are the medians of the recognition rates obtained by SFL-LBP and LBP on all face databases. In our experiments, the significance level is set to 0.05. From the test results in Table 8, it can be found that the *p*-values obtained by all pairwise Wilcoxon rank sum tests are less than the significance level, which indicates that the null hypotheses are rejected in all pairwise tests and the proposed algorithm significantly outperforms the other algorithms.

**Table 8:** The *p*-values of the pairwise one-tailed Wilcoxon rank sum tests.

Algorithms	SRC	LRC
SFL-LBP(*S* = *r* = 3)vs.LBP	0.0015	0.00059
SFL-LBP(*S* = *r* = 5)vs.LBP	0.000053	0.0016
SFL-LBP(*S* = *r* = 7)vs.LBP	0.0013	0.0015
SFL-LBP(*S* = *r* = 3)vs. LGBP	0.0016	0.000043
SFL-LBP(*S* = *r* = 5)vs. LGBP	0.00011	0.00028
SFL-LBP(*S* = *r* = 7)vs. LGBP	0.0021	0.00048
SFL-LBP(*S* = *r* = 3)vs. GVLBP	0.0067	0.00089
SFL-LBP(*S* = *r* = 5)vs. GVLBP	0.00027	0.0154
SFL-LBP(*S* = *r* = 7)vs. GVLBP	0.0038	0.0209
SFL-LBP(*S* = *r* = 3)vs. DSNPE	0.0062	0.000028
SFL-LBP(*S* = *r* = 5)vs. DSNPE	0.00012	0.00027
SFL-LBP(*S* = *r* = 7)vs. DSNPE	0.0035	0.0003
SFL-LBP(*S* = *r* = 3)vs. MNSMC	0.0264	0.000011
SFL-LBP(*S* = *r* = 5)vs. MNSMC	0.0006	0.00007
SFL-LBP(*S* = *r* = 7)vs. MNSMC	0.0106	0.00014
SFL-LBP(*S* = *r* = 3)vs. UDSPP	0.0051	0.000092
SFL-LBP(*S* = *r* = 5)vs. UDSPP	0.0011	0.0015
SFL-LBP(*S* = *r* = 7)vs. UDSPP	0.0079	0.0029
SFL-LBP(*S* = *r* = 3)vs.IFL-LBP(*S* = *r* = 3)	0.008	0.0195
SFL-LBP(*S* = *r* = 5)vs.IFL-LBP(*S* = *r* = 5)	0.0089	0.0062
SFL-LBP(*S* = *r* = 7)vs.IFL-LBP(*S* = *r* = 7)	0.01	0.0155

## Conclusions

This paper presents a filter learning algorithm for representation based face recognition. Due to the objective function of our proposed algorithm is specially designed to reduce the within-class representation residual and enlarge the between-class representation residual of faces' local descriptors, it is more suitable for the representation based classifiers than other algorithms. In the experiments, five public face databases are utilized to evaluate our algorithm. Through comparing our algorithm with other state-of-the-art algorithms using two well-known representation based classifiers, the effectiveness and advantage of our algorithm are demonstrated.

## References

[1] Jain AK, Li SZ. Handbook of face recognition: Springer; 2005.

[2] Wright J, Yang AY, Ganesh A, Sastry SS, Ma Y. Robust face recognition via sparse representation. Pattern Analysis and Machine Intelligence, IEEE Transactions on. 2009;31(2):210–27. 10.1109/TPAMI.2008.79 19110489

[3] Naseem I, Togneri R, Bennamoun M. Linear regression for face recognition. Pattern Analysis and Machine Intelligence, IEEE Transactions on. 2010;32(11):2106–12. 10.1109/TPAMI.2010.128 20603520

[4] Gao S, Tsang IW-H, Chia L-T. Kernel sparse representation for image classification and face recognition Computer Vision–ECCV 2010: Springer; 2010 p. 1–14.

[5] Wang J, Yang J, Yu K, Lv F, Huang T, Gong Y. Locality-constrained linear coding for image classification. Computer Vision and Pattern Recognition (CVPR), 2010 IEEE Conference on; 2010: IEEE.

[6] Yang M, Zhang L, Yang J, Zhang D. Robust sparse coding for face recognition. Computer Vision and Pattern Recognition (CVPR), 2011 IEEE Conference on; 2011: IEEE.

[7] Deng W, Hu J, Guo J. Extended SRC: Undersampled face recognition via intraclass variant dictionary. Pattern Analysis and Machine Intelligence, IEEE Transactions on. 2012;34(9):1864–70. 10.1109/TPAMI.2012.30 22813959

[8] Mi J-X, Liu J-X. Face recognition using sparse representation-based classification on k-nearest subspace. PloS one. 2013;8(3):e59430 doi: 10.1371/journal.pone.0059430 23555671 10.1371/journal.pone.0059430 PMC3608681

[9] Turk M, Pentland A. Eigenfaces for recognition. Journal of cognitive neuroscience. 1991;3(1):71–86. doi: 10.1162/jocn.1991.3.1.71 23964806 10.1162/jocn.1991.3.1.71

[10] Scholkopft B, Mullert K-R. Fisher discriminant analysis with kernels. Neural networks for signal processing IX. 1999;1:1.

[11] Yang J, Zhang D, Frangi AF, Yang J-y. Two-dimensional PCA: a new approach to appearance-based face representation and recognition. Pattern Analysis and Machine Intelligence, IEEE Transactions on. 2004;26(1):131–7. 10.1109/tpami.2004.1261097 15382693

[12] Pentland A, Moghaddam B, Starner T. View-based and modular eigenspaces for face recognition. Computer Vision and Pattern Recognition, 1994 Proceedings CVPR'94, 1994 IEEE Computer Society Conference on; 1994: IEEE.

[13] Howland P, Park H. Generalizing discriminant analysis using the generalized singular value decomposition. Pattern Analysis and Machine Intelligence, IEEE Transactions on. 2004;26(8):995–1006. 10.1109/TPAMI.2004.46 15641730

[14] Ye J, Li Q. A two-stage linear discriminant analysis via QR-decomposition. Pattern Analysis and Machine Intelligence, IEEE Transactions on. 2005;27(6):929–41. 10.1109/TPAMI.2005.110 15943424

[15] Belhumeur PN, Hespanha JP, Kriegman DJ. Eigenfaces vs. fisherfaces: Recognition using class specific linear projection. Pattern Analysis and Machine Intelligence, IEEE Transactions on. 1997;19(7):711–20.

[16] Roweis ST, Saul LK. Nonlinear dimensionality reduction by locally linear embedding. Science. 2000;290(5500):2323–6. 11125150 10.1126/science.290.5500.2323

[17] Tenenbaum JB, De Silva V, Langford JC. A global geometric framework for nonlinear dimensionality reduction. Science. 2000;290(5500):2319–23. 11125149 10.1126/science.290.5500.2319

[18] Belkin M, Niyogi P. Laplacian eigenmaps for dimensionality reduction and data representation. Neural computation. 2003;15(6):1373–96.

[19] Lei Z, Yi D, Li SZ, Discriminant image filter learning for face recognition with local binary pattern like representation. Computer Vision and Pattern Recognition (CVPR), 2012 IEEE Conference on; 2012: IEEE.

[20] Mikolajczyk K, Schmid C. A performance evaluation of local descriptors. Pattern Analysis and Machine Intelligence, IEEE Transactions on. 2005;27(10):1615–30. 10.1109/TPAMI.2005.188 16237996

[21] Penev PS, Atick JJ. Local feature analysis: A general statistical theory for object representation. Network: computation in neural systems. 1996;7(3):477–500.

[22] Ojala T, Pietikäinen M, Harwood D. A comparative study of texture measures with classification based on featured distributions. Pattern recognition. 1996;29(1):51–9.

[23] Shan S, Gao W, Chang Y, Cao B, Yang P. Review the strength of gabor features for face recognition from the angle of its robustness to mis-alignment. Pattern Recognition, 2004 ICPR 2004 Proceedings of the 17th International Conference on; 2004: IEEE.

[24] Zhang W, Shan S, Gao W, Chen X, Zhang H, Local gabor binary pattern histogram sequence (lgbphs): A novel non-statistical model for face representation and recognition. Computer Vision, 2005 ICCV 2005 Tenth IEEE International Conference on; 2005: IEEE.

[25] Lei Z, Liao S, Pietikäinen M, Li SZ. Face recognition by exploring information jointly in space, scale and orientation. Image Processing, IEEE Transactions on. 2011;20(1):247–56. 10.1109/TIP.2010.2060207 20643604

[26] Zhang B, Shan S, Chen X, Gao W. Histogram of gabor phase patterns (hgpp): A novel object representation approach for face recognition. Image Processing, IEEE Transactions on. 2007;16(1):57–68. 10.1109/tip.2006.884956 17283765

[27] Zhao S, Gao Y, Zhang B, editors. Sobel-lbp. Image Processing, 2008 ICIP 2008 15th IEEE International Conference on; 2008: IEEE.

[28] Min R, Dugelay J-L. Improved combination of LBP and sparse representation based classification (SRC) for face recognition. Multimedia and Expo (ICME), 2011 IEEE International Conference on; 2011: IEEE.

[29] Huang M-W, Wang Z-w, Ying Z-L. A new method for facial expression recognition based on sparse representation plus LBP. Image and Signal Processing (CISP), 2010 3rd International Congress on; 2010: IEEE.

[30] Kang C, Liao S, Xiang S, Pan C. Kernel sparse representation with local patterns for face recognition. Image Processing (ICIP), 2011 18th IEEE International Conference on; 2011: IEEE.

[31] Lee H, Chung Y, Kim J, Park D. Face image retrieval using sparse representation classifier with gabor-lbp histogram Information Security Applications: Springer; 2011 p. 273–80.

[32] Yan S, Xu D, Zhang B, Zhang H-J, Yang Q, Lin S. Graph embedding and extensions: a general framework for dimensionality reduction. Pattern Analysis and Machine Intelligence, IEEE Transactions on. 2007;29(1):40–51. 10.1109/TPAMI.2007.12 17108382

[33] Ojala T, Pietikäinen M, Mäenpää T. Multiresolution gray-scale and rotation invariant texture classification with local binary patterns. Pattern Analysis and Machine Intelligence, IEEE Transactions on. 2002;24(7):971–87.

[34] Ahonen T, Hadid A, Pietikainen M. Face description with local binary patterns: Application to face recognition. Pattern Analysis and Machine Intelligence, IEEE Transactions on. 2006;28(12):2037–41. 10.1109/TPAMI.2006.244 17108377

[35] Zhu M, Martinez AM. Subclass discriminant analysis. Pattern Analysis and Machine Intelligence, IEEE Transactions on. 2006;28(8):1274–86. 10.1109/TPAMI.2006.172 16886863

[36] Lei Z, Pietikainen M, Li SZ. Learning discriminant face descriptor. Pattern Analysis and Machine Intelligence, IEEE Transactions on. 2014;36(2):289–302. 10.1109/TPAMI.2013.112 24356350

[37] Lu G-F, Jin Z, Zou J. Face recognition using discriminant sparsity neighborhood preserving embedding. Knowledge-Based Systems. 2012;31:119–27.

[38] Chen Y, Li Z, Jin Z. Feature extraction based on maximum nearest subspace margin criterion. Neural processing letters. 2013;37(3):355–75.

[39] Chen Z, Huang W, Lv Z. Towards a face recognition method based on uncorrelated discriminant sparse preserving projection Multimedia Tools and Applications. 2015:1–15.

[40] Georghiades A. Yale face database. Center for computational Vision and Control at Yale University. Available: http://cvc.yale.edu/projects/yalefaces/yalefa. 1997.

[41] Martinez AM. The AR face database. CVC Technical Report. 1998;24.

[42] Sim T, Baker S, Bsat M. The CMU pose, illumination, and expression (PIE) database. Automatic Face and Gesture Recognition, 2002 Proceedings Fifth IEEE International Conference on; 2002: IEEE.

[43] Huang GB, Ramesh M, Berg T. Learned-Miller E. Labeled faces in the wild: A database for studying face recognition in unconstrained environments. Technical Report 07–49, University of Massachusetts, Amherst, 2007.

[44] Xu Y, Zhong A, Yang J, Zhang D. Bimodal biometrics based on a representation and recognition approach. Optical Engineering. 2011;50(3):037202–7.

[45] Raudys SJ, Jain AK. Small sample size effects in statistical pattern recognition: Recommendations for practitioners. IEEE Transactions on Pattern Analysis & Machine Intelligence. 1991;(3):252–64.

